# An evaluation of the cost of human papilloma virus (HPV) vaccine delivery in Zambia

**DOI:** 10.1186/s12879-024-09222-2

**Published:** 2024-04-02

**Authors:** Moses C Simuyemba, Chitalu M Chama-Chiliba, Abson Chompolola, Aaron Sinyangwe, Abdallah Bchir, Gilbert Asiimwe, Carla Chibwesha, Felix Masiye

**Affiliations:** 1 Department of Community and Family Medicine, University of Zambia School of Public Health, Ridgeway Campus, Lusaka, Zambia; 2 Department of Economics, School of Humanities and Social Sciences, University of Zambia, Lusaka, Zambia; 3 Monastir Medical School, University of Monastir, Monastir, Tunisia; 4 The Vaccine Alliance, Gavi, Geneva, Switzerland; 5 University of North Carolina Global Women’s Health, Chapel Hill, United States of America

**Keywords:** HPV vaccine, Human papilloma virus, Zambia, Cost of HPV vaccination, Gavi, HPV vaccine in Africa

## Abstract

**Background:**

Human papillomavirus (HPV) is a common sexually transmitted infection and the leading cause of cervical cancer. The HPV vaccine is a safe and effective way to prevent HPV infection. In Zambia, the vaccine is given during Child Health Week to girls aged 14 years who are in and out of school in two doses over two years. The focus of this evaluation was to establish the cost to administer a single dose of the vaccine as well as for full immunisation of two doses.

**Methods:**

This work was part of a broader study on assessing HPV programme implementation in Zambia. For HPV costing aspect of the study, with a healthcare provider perspective and reference year of 2020, both top-down and micro-costing approaches were used for financial costing, depending on the cost data source, and economic costs were gathered as secondary data from Expanded Programme for Immunisation Costing and Financing Project (EPIC), except human resource costs which were gathered as primary data using existing Ministry of Health salary scales and reported time spent by different health cadres on activities related to HPV vaccination. Data was collected from eight districts in four provinces, mainly using a structured questionnaire, document reviews and key informant interviews with staff at national, provincial, district and health facility levels. Administrative coverage rates were obtained for each district.

**Results:**

Findings show that schools made up 53.3% of vaccination sites, community outreach sites 30.9% and finally health facilities 15.8%. In terms of coverage for 2020, for the eight districts sampled, schools had the highest coverage at 96.0%. Community outreach sites were at 6.0% of the coverage and health facilities accounted for only 1.0% of the coverage. School based delivery had the lowest economic cost at USD13.2 per dose and USD 28.1 per fully immunised child (FIC). Overall financial costs for school based delivery were US$6.0 per dose and US$12.4 per FIC. Overall economic costs taking all delivery models into account were US$23.0 per dose and US$47.6 per FIC. The main financial cost drivers were microplanning, supplies, service delivery/outreach and vaccine co-financing; while the main economic cost drivers were human resources, building overhead and vehicles. Nurses, environmental health technicians and community-based volunteers spent the most time on HPV related vaccination activities compared to other cadres and represented the greatest human resource costs.

**Conclusions:**

The financial cost of HPV vaccination in Zambia aligns favourably with similar studies conducted in other countries. However, the economic costs appear significantly higher than those observed in most international studies. This discrepancy underscores the substantial strain placed on healthcare resources by the program, a burden that often remains obscured. While the vaccine costs are currently subsidized through the generous support of Gavi, the Vaccine Alliance, it’s crucial to recognize that these expenses pose a considerable threat to long-term sustainability. Consequently, countries such as Zambia must proactively devise strategies to address this challenge.

**Supplementary Information:**

The online version contains supplementary material available at 10.1186/s12879-024-09222-2.

## Background

Human papillomavirus (HPV) is a common sexually transmitted infection that can cause a range of health problems, including genital warts and certain types of cancer [1–3]. The HPV vaccine is a safe and effective way to prevent HPV infection and the health problems it can cause [1, 2, 4]. 

Since 2019, the HPV vaccine in Zambia is provided through the country’s Expanded Programme on Immunisation (EPI), once a year during the Child Health Week (CHW) in the month of June, targeting girls aged 14 years who are in and out of school. The overall target for the programme is girls aged 9 to 14, but so far only the 14-year-olds have been vaccinated owing to inadequate doses of HPV vaccine being available on the global market and thus Gavi could only secure a limited number of vaccines [5–7]. 

The Zambian EPI prioritized the vaccination of 14-year-olds, as it represented their final opportunity to receive the vaccine before surpassing the age limit. HPV vaccine distribution in Zambia employs various channels, including health facilities, schools, and community outreach sites, to ensure widespread accessibility and uptake. The primary focus of social mobilization and messaging revolves around maximizing vaccination rates among schoolgirls, with health facilities and outreach sites catering to those not enrolled in school. The Ministry of Health collaborates with international and non-governmental partners to facilitate vaccine accessibility, promote public awareness of vaccination’s significance, and dispel vaccine-related myths and misconceptions.

Gavi, the Vaccine Alliance, played a vital role in supporting HPV vaccine delivery by providing funding to the country’s national immunization program to help cover the costs of purchasing and distributing the vaccine [5]. Gavi has also provided technical assistance and capacity building to help the Ministry of Health implement effective strategies for increasing vaccine uptake, and has supported the training of health care workers to administer the vaccine safely and effectively.

In recommending HPV vaccine introduction the Zambia Immunisation Technical Advisory Group, ZITAG, used cost and cost-effectiveness studies from Brazil, Canada, the United Kingdom, and Tanzania [6, 8]. The transferability of such findings to the Zambian context was arguable and such decisions were often heavily influenced by global level directives made by donors (Wilkinson, Sculpher et al. 2016) [6]. 

A PATH study during the demonstration project estimated the financial cost at between US$9.98 and US$10.40 per fully immunised girl (PATH 2014) [9]. A Gavi Full Country Evaluation (FCE) report stated that the financial and programmatic sustainability implications of introducing HPV vaccine nationally were not thoroughly assessed using local evidence and recommended that the Ministry of Health develop clear policy and guidelines for purposes of economic evaluation of new vaccine introductions [6]. As Zambia proceeds with the national introduction the question is now centred around understanding the cost of vaccination for a nationwide programme compared to a demonstration project in only one province. The objective of this paper was to establish the cost to administer as single dose as well as to get a girl fully vaccinated across the three delivery platforms being utilised, namely school, community outreach and health facility, now that HPV vaccination is nationwide. The paper also highlights an approach that can be taken to apportion costs across different vaccine delivery platforms in settings where such apportionment may be challenging.

## Methods

For HPV costing, a healthcare provider perspective was used and both top-down and micro-costing approaches were exploited, depending on the source of the cost data, to estimate the financial and economic costs of implementing HPV vaccination using the delivery model of child health week [10]. Primary data was collected for financial costing as well as the economic cost of human resources, and secondary data from the updated Expanded Programme for Immunisation Costing and Financing Project (EPIC) study was used for the other economic costs [11]. Primary data was collected between July and September 2020. The EPIC study is a comprehensive study aimed at assessing routine immunization and new vaccines costs with a view to optimizing the costs and financial sustainability of immunization programs worldwide, and it included Zambia as one of its focus countries [11]. Micro-costing is a method of estimating the costs of delivering a specific intervention or program at a detailed level and involves identifying and quantifying all of the resources and activities that are needed to deliver the intervention or program, and then assigning a cost to each of these elements. Top-down costing is a high-level approach that estimates the overall cost of a program or service based on aggregated data and in this case, this was data from national level budgets of the Expanded Programme on Immunisation. Table 1 summarises the resource input categories. Some primary data was gathered from 2019 and others from 2020 vaccination week and the 2019 costs were inflated to 2020 equivalent at 9.15% inflation rate [12]. Costs were gathered in both USD and ZMW and exchange rate of ZMW18.36 was used for 2020, based on Bank of Zambia exchange rate data [13]. 

**Table 1:** Micro-costing for HPV vaccination in Zambia – Resource Input Categories

Cost category	Resource Input	Notes	Data source
1. Micro-planning	Per diem, transport allowances, other allowances, fuel, venue hire, lodging, refreshments and meals, conference supplies, equipment hire, printing health cards, registers, tally sheets, distribution costs	Microplanning costs were gathered at for national and district levels	Excel questionnaire and EPI documents and budgets
2. Training	Facilitators, resource persons, trainers, and participant per diems, support staff per diems, transport allowances, other allowances, venue hire, lodging, refreshments, conference supplies, equipment hire, printing of health cards, tally sheets and registers, other materials	Training at national and district levels as well as health facility level	Excel questionnaire and EPI documents and budgets
3. Service delivery	Per diem to vaccinators, project management and support staff; fuel and lubricants, Vehicle maintenance and/or repair costs, vehicle hire, other vehicle costs, waste management supplies, M&E, carriers, ice packs, banners, other costs, laboratory supplies	Service delivery applied to the vaccination week and to all delivery models (school, health facility, community outreach)	Excel questionnaire and EPI documents and budgets
4. Vaccines and supplies	Doses received 2019 and 2020; Doses used in 2019 and 2020	Vaccine costs related to co-financing obligation at USD 0.55 per dose as Gavi bore a major part of the cost	EPI documents and budgets
5. Cold chain specifically purchased for HPV vaccine	Purchase price, maintenance, electricity, fuel	Cold chain specifically for HPV vaccine in 2019 and 2020	EPI documents and budgets
6. Waste management	Safety boxes, plastic bags, gloves, other protective gear, sterilisation chemicals, other materials	Waste management supplies specifically bought for HPV vaccine	Excel questionnaire
7. Other Cold chain used for HPV vaccine	Purchase price, electricity, proportion used for HPV vaccine, maintenance, fuel	All economic costs obtained from EPIC study and were outlined as cost per facility. Thus the costs obtained were multiplied by number of facilities and then apportioned to HPV as 50% in that on average health facilities reported using half the child health week for HPV vaccination activities.	EPIC Study
8. Other equipment	Total and proportion used for HPV vaccine		
9. Building overhead	Total and proportion used for HPV vaccine		
10. Vehicles	Total and proportion used for HPV vaccine		
11. Vehicle maintenance	Total and proportion used for HPV vaccine		
12. Human Resources	Receiving training, conducting training, preparation and planning, Child Health Week activities, Mop-up activities after child health week, supportive supervision, data collection and reporting, community mobilisation, surveillance relating to HPV. Cadres included District Director of Health, Medical Doctor, Medical Licenciate, Health Information Officer, MCH coordinator, Health promotion officer, clinical officer, planner, M&E officer, Accountant, Logistician, Environmental Health Technologist, Registered Nurse/Midwife, Driver, Community health assistant, Community Based Volunteers, Nursing officer, Adolescent Health focal point person, Nutritionist, Nursing Officer MCH, HMIS officer, Others	Human resource data collection focused on time spent on the activities listed by each health cadre and apportioning costs to this time based on the salary scale of the cadre.	EPI documents and budgets

Note: Resource-input categories utlised were in line with costing utlised by the Ministry of Health in Zambia

Data for these costs was collected at both national and subnational levels mainly using a structured questionnaire, supplemented by document reviews (HPV microplans, reports, budgets) and key informant interviews with staff at national, provincial, district and health facility levels. A sample of eight districts in four provinces was included in order to be more representative than the earlier demonstration phase costing. The sample for the subnational level built on the EPI Costing and Financing Project (EPIC) sample with inclusion of an additional province in consultation with the Ministry of Health [14]. These were Lusaka province (Lusaka and Chongwe districts); Copperbelt province (Ndola and Lufwanyama districts); Central province (Kabwe and Mkushi districts) and Muchinga province as the addition (Mpika and Chinsali districts). Expenditure data on all key activities, including planning, social mobilization, vaccines and other supplies, service delivery, supervision, data collection and compilation, was included. In addition, the data collection tool included information on number of health facilities, outreach sites as well as schools that were utilised in the HPV vaccination exercise, as well as coverage data across all these sites for each district sampled. For all these costs there was specific emphasis on where the costs were incurred with reference to the delivery models of school, community outreach and health facility.

Data analysis varied according to data source and level of data. For national level data, costs were allocated to each district if they were budgeted as such as the budget matched the expenditure as illustrated by relevant personnel providing the data. Costs that were not specific to a district were allocated by dividing the total cost by the number of districts in the country. For data collected from the eight sampled districts, total costs for all eight districts and average costs per district were calculated. The total costs were apportioned to each delivery model (school, health facility and outreach) based on the proportion of vaccination sites utilised for each model. This approach was used as it was generally not possible to allocate specific costs to each delivery model due to the manner in which district level budgets were made without such specific reference to each delivery model. Economic costs were gathered from EPIC study data. Univariate and multivariate sensitivity analysis was carried out to assess the output with cost per dose as the dependent variable and all cost inputs listed in Table 2 as independent variables, adjusting them between 1 and 10% [15–17].

**Table 2:** Costs attributable to HPV Vaccination in Zambia, 2020

COSTS	Sum	Average/District	Dose 2 Costs (46%)	% of total costs
Financial costs				
**Planning and Training**				
*Microplanning*	47,595	5,949	22,018	4.7%
*Training*	23,266	2,908	10,763	2.3%
***Orientation***				
Orientation/Training	8,424	1,053	3,897	0.8%
***Social Mob and Service Delivery***				
*Social Mobilisation*	5,535	692	2,561	0.5%
*Service delivery/Outreach*	48,162	6,020	22,281	4.7%
*Supervision & M&E*	4,602	575	2,129	0.5%
*communication*	436	54	202	0.0%
***Vaccines and Supplies***				
*Supplies (safety boxes, needles, syringes etc.)*	77,909	9,739	36,043	7.6%
*Vaccines Co-finance*	25,902	3,238	11,983	2.5%
*Health Cards + Registers + Tally Sheets + Other materials*	3,224	403	1,491	0.3%
*Distribution*	956	119	442	0.1%
*Other costs*	982	123	454	0.1%
***Delivery model specific costs***				
*School Outreach*	9,545	1,193	4,416	0.9%
*Health Facility*	4,308	539	1,993	0.4%
*Community Outreach*	5,355	669	2,477	0.5%
***Economic costs apportioned***				
*Cold chain maintenance and utilities*	19,838	2,480	9,177	1.9%
*Sterilisation and Waste management*	37,304	4,663	17,159	3.6%
*Cold chain equipment*	60,332	7,541	27,911	5.9%
*Cold Chain Energy Costs*	32,373	4,046	14,891	3.2%
*Other equipment*	86,001	10,750	39,786	8.4%
*Building overhead*	121,317	15,165	56,124	11.9%
*Vehicles*	105,458	13,182	48,787	10.3%
*vehicle maintenance*	19,293	2,412	8,925	1.9%
*Human Resources*	274,220	34,277.50	126,860	26.8%
***TOTAL (USD) including HR***	**1,022,337**	**119,082**	**472,957**	100.0%
**Financial Cost**	**266,201**	**33,275**	**122,452**	

Note: All costs derived from district data collected in eight districts as sampled, except economic costs which were derived from EPIC study.

The total costs was apportioned to each delivery model based on the data in Table 3 on the proportion of delivery sites for each model. This was divided by the coverage (dose 1 and dose 2) for each model as outlined in Table 4 to arrive at the cost per dose. For overall costs and across delivery models, the cost per fully immunised child was arrived at by dividing total costs by number of fully immunised girls for each delivery models.

## Findings

Table 3 provides details of the three delivery models used in Zambia for HPV vaccine delivery across the eight sampled districts. Schools made up more than half the sites for vaccination, followed by community outreach sites and finally health facilities. This was used as a basis for apportioning costs later.

**Table 3:** HPV Delivery models in Zambia

Delivery model	Number of sites	Percentage
Health facilities	327	15.8%
Community outreach sites	641	30.9%
Schools	1108	53.3%
**Total**	**2076**	**100.0%**

Note: Data derived from district data collected in eight districts as sampled

In terms of coverage for 2020, for the eight districts sampled, schools had the highest coverage, averaging 93% of the coverage across first and second doses. Community outreach sites were at 6.0% of the coverage and health facilities accounted for only 1.0% of the coverage as shown in Table 3.

Table 4 gives details of the costs attributable to HPV vaccination in Zambia. The table shows that schools represented the major high number of administered doses for both first and second doses, followed by outreach sites and finally health facilities had vaccine doses adminsitered. Second dose (full immunisation) accounted for 46.3% of all doses administered in the sampled districts in 2020.

**Table 4:** HPV Vaccine Doses administered 2020

Dose	Doses administered	Percentage by site	Percentage of total doses administered
**First Dose**	22,896		53.7%
*Schools*	21,874	95.5%	
*Community Outreach Sites*	947	4.1%	
*Health Facilities*	76	0.3%	
**Second Dose**	**21,460**		**46.3%**
*Schools*	19,390	90.4%	
*Community Outreach Sites*	1,706	7.9%	
*Health Facilities*	364	1.7%	
**All Doses Total**	**44,356**		**100%**
*Schools*	41,264	93.0%	
*Community Outreach Sites*	2,653	6.0%	
*Health Facilities*	440	1.0%	

Note: Data derived from district data collected in eight districts as sampled

As illustrated in Table 2, human resource-related costs remain the largest costs associated with the EPI programme and HPV vaccination is no exception. In the table above, most of the costs, other than vaccine costs, related to staff allowances, per diem and fuel (microplanning, training, service delivery and outreach). Enrolled Nurse/Midwife position accounted the largest proportion of time spent on HPV vaccination by all cadres at 27.4%. This was followed by Registered Nurse/Midwife at 24.7%, Environmental Health Technologist (EHT) at 12.2%, Community based volunteers (CBV) at 11.2% and clinical officers at 6.6%. Social mobilisation and supervision/monitoring and evaluation were highly underfunded, according to key informants who stated that it was inadequate.

Finally, Table 5 provides the calculated costs based on the foregoing tables. School based delivery had the lowest cost economic cost at USD13.2 per dose and USD 28.1 per fully immunised child. It also had the lowest financial cost at US$3.4 per dose and US$7.3 per FIC. Total financial costs across all delivery platforms were US$6.0 per dose and US$12.4 per fully immunised child. Overall economic costs taking all delivery models into account were US$23.0 per dose and US$47.6 per FIC.

**Table 5:** HPV vaccinations cost per dose and perf fully immunised child in Zambia

Costs apportioned to delivery model	Cost (USD)	Doses administered	Cost per dose (USD)	Cost per FIC (2 doses, USD)
**Economic Cost**				
TOTAL	1,022,337 (100%)	44,356	23.0	47.6
Health facilities	161,033 (15.8% of total)	440	365.7	441.8
Outreach	315,664 (30.9% of total)	2,652	119.0	185.0
Schools	545,640 (53.4% of total)	41,264	13.2	28.1
**Financial Cost**				
TOTAL	266,202 (100%)	44,356	6.0	12.4
Health facilities	41,931 (15.8% of total)	440	95.2	115.0
Outreach	82,194 (30.9% of total)	2,652	31.0	48.2
Schools	142,077 (53.4% of total)	41,264	3.4	7.3

Note: Calculations are based on coverage and costs derived from eight sampled districts and EPIC study

Sensitivity analysis showed that health facility delivery is most affected by changes in costs, mainly due to the low coverage achieved there. Schools were least sensitive to changes in costs due to higher coverage.

## Discussion

The cost of administering HPV vaccine across different delivery platforms in Africa is a critical factor in determining the success of HPV vaccination programs [17–21]. School-based delivery has been shown to be the most economical method for delivering the vaccine, as it requires minimal resources when we consider the cost per FIC, and it can be implemented quickly with higher coverage in most countries [18, 20, 22, 23]. The findings of this evaluation support this as school based HPV vaccine delivery had the highest coverage and lowest cost per dose. According to key informants, orientation and social mobilisation efforts were highly focused on the schools and less so on health facility access and outreach. This may disadvantage out-of-school girls in terms of accessing the vaccine [18]. Additionally, personnel based at health facilities were not so focused on HPV vaccination, despite them being mandated to do so. Both health facility and community outreach were meant to help with vaccinating out-of-school girls, a hard-to-reach population [18]. In Cambodia and Zimbabwe, out-of-school girls identified by community health workers were invited to come to schools for vaccination, But the effectiveness of this approach could not be shown, illustrating the enormity of the challenge [20, 24]. 

Health facility-based delivery is also potentially an efficient option, although the cost of facility-based delivery is higher due to the need for additional equipment and personnel and usually lower coverage in most countries that have implemented this approach [18, 20]. Community-based delivery is an important option for reaching rural and hard-to-reach populations, but the cost of community-based delivery is often higher than school-based or health facility-based delivery due to logistical challenges, higher economic costs, and the need for additional resources to do with social mobilisation, for example [20]. Cost-effectiveness studies have shown that the cost of delivering the HPV vaccine through different delivery platforms in Africa can vary significantly, depending on the type of delivery platform, the population being targeted, and the resources available [18]. 

A study of costs of HPV vaccination in Gavi supported countries using the World Health Organization Cervical Cancer Prevention and Control Costing Tool found the average economic cost per dose to have been US$19.98, and US$8.74 as the financial cost across one year demonstration projects [17]. The economic cost of US$23.0 per dose for Zambia from this study is compared to other available studies in the African countries [18–24]. In these studies, the highest economic cost per dose at US$45.0 was in Zimbabwe and the lowest at US$3.09 was in Mwanza district in Tanzania, although the national average of US$10.62 for Tanzania may be a better comparison. Senegal had an economic cost of US$12.24 and financial cost of US$ 7.75, whereas Mozambique had an economic cost of US$24 [25]. 

In terms of financial costs the main cost drivers in Zambia were supplies (e.g. syringes, needles, safety boxes etc.) as well as costs associated with per diems and allowances for staff and community workers and volunteers (relating to microplanning, training, orientation, social mobilisation and monitoring and evaluation as well as actually vaccination activities). When we consider economic costs human resource costs were the largest, followed by buildings and vehicles and then cold chain equipment and maintenance.

Human resource related costs in terms of per diems, allowances and salaries were the largest cost drivers for most of the countries whose studies have been included here [18–24]. As shown in the results, nurses (both enrolled and registered) made up over half of the total time spent by all cadres on HPV immunisation activities, with the next most utilised cadres being CBVs. The EHTS took up roles related to social mobilisation and community outreach due to lack of specific cadres employed for such work. M&E Officer, Accountant, driver and community health assistants were additional positions that dedicated the most time ranging between 2% and 3.5%. Planning for HPV vaccination in Zambia thus needs to prioritise these critical positions in terms or resource allocation and support.

Demonstration project data for Gavi supported countries showed that social mobilisation and service delivery were the largest cost drivers, but this did not take into account economic costs [18]. However, this is not usually the case beyond demonstration projects as social mobilisation was usually heavily funded for demonstration projects but not as well funded during the actual national implementation, as have been the case in Zambia. In fact when funds are limited, activities like social mobilisation become the likely victims of reduced allocation of funds. Monitoring and evaluation is another area that is often under-funded during national rollout when funds are more limited.

One of the main drivers of cost for HPV vaccination in Africa is the cost of the vaccine itself. However, many Gavi supported countries do not bear the entire vaccine cost. Gavi, has negotiated vaccine prices with manufacturers and accessed the bivalent vaccine at US$4.50 and the quadrivalent vaccine at US$4.60 per dose [17]. In Zambia, the government only put up US$0.55 per dose as their co-finance contribution to the Gavi support. Without Gavi support, vaccine costs become a major cost driver and barrier to HPV vaccination programmes in Africa. Strategies thus need to be put in place in countries like Zambia to cater for how the vaccine cost will be funded once Gavi support ceases.

There are several methodological challenges and complexities associated with apportioning costs across HPV Vaccination delivery platforms which form part of the limitations of this study [26, 27]. Data availability and quality is one such challenge as obtaining accurate and detailed cost data can be challenging, particularly in low-resource settings where financial and administrative systems may be less developed. Incomplete or missing data can lead to inaccurate cost estimates [27, 28]. In this study, for example, supporting documents for financial data provided were not always available there was also some likelihood of recall bias given time from activity to this data collection. Secondly, allocating costs for shared resources, such as personnel, facilities, and equipment, can be complex. Different methods, such as direct allocation or step-down allocation, may produce varying results, and the choice of method can significantly impact cost estimates [26, 28]. This study settled on using EPIC data for most of the economic costs, other than human resources.

Thirdly, The costs of delivering the HPV vaccine may vary depending on factors such as geography, population density, and infrastructure. This variability can make it challenging to generalize cost estimates across different delivery platforms and settings. Lastly, the costs of HPV vaccination programs can change over time as the program scales up, achieves economies of scale, or faces changes in vaccine prices as well as fluctuations in exchange rates [29]. Accounting for these changes in cost estimates is crucial for accurately assessing the cost-effectiveness of the program [30, 31]. 

The problem of cost allocation across delivery platforms if further compounded by having multiple sources of funds, including government monthly grants and special funding from donors such as Gavi, and Unite Nations agencies which may not be earmarked for any one particular delivery platform.

## Conclusion

The financial cost of HPV vaccination in Zambia aligns favourably with similar studies conducted in other countries. However, the economic costs appear significantly higher than those observed in most international studies. This discrepancy underscores the substantial strain placed on healthcare resources by the program, a burden that often remains obscured. While the vaccine costs are currently subsidized through the generous support of Gavi, the Vaccine Alliance, it’s crucial to recognize that these expenses pose a considerable threat to long-term sustainability. Consequently, countries such as Zambia must proactively devise strategies to address this challenge.

### Electronic supplementary material

Below is the link to the electronic supplementary material.

Supplementary Material 1

Supplementary Material 2

Supplementary Material 3

## Data Availability

Data is available on request from the corresponding author.

## Abbreviations

CBV: Community–based volunteer
CHW: Child Health Week
EHT: Environmental Health Technologist
EPI: Expanded Programme on Immunisation
EPIC: Expanded Programme for Immunisation Costing and Financing Project
FCE: Full Country Evaluation
FIC: Fully Immunised Child
HPV: Human Papilloma Virus
M&E: Monitoring and Evaluation
MOH: Ministry of Health
ZITAG: Zambia Immunisation Technical Advisory Group
