# Supplementary material for: An evaluation of the cost of human papilloma virus (HPV) vaccine delivery in Zambia
Source: BMC Infect Dis. 2024 Apr 2;24:369. doi: 10.1186/s12879-024-09222-2 (PMC10986043; doi:10.1186/s12879-024-09222-2)
Supplement: Supplementary file 2 — Supplementary Material 2 [file 12879_2024_9222_MOESM2_ESM.doc]

**
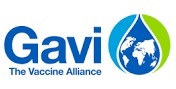
**
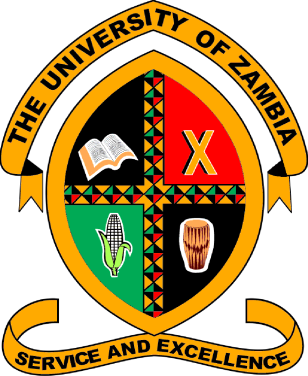


**Key informant interviews**

**Questions on HPV Implementation and Routinisation**

**EPI partners and MOH national and subnational levels**

**Evaluation of Immunisation Programme Sustainability in Zambia**

**Background information**

Date of interview (day/month/year): ________________________

Role in EPI: ______________________________________________

Number of years involved with EPI: _____________

Beginning time of interview: __________

Name of the interviewer: ______________________________________________

Name of the note taker: _______________________________________________

**Respondents:** Key informants from MOH, Cooperating Partners and other EPI Partners at national, provincial and district levels

**Introduction**

Zambia embarked on an HPV demonstration project from 2013 to 2017. This demonstration project generated a lot of lessons for the country. These lessons were utilised to plan for national introduction. A first dose of HPV vaccine was delivered to girls aged 14 years in 2019 with a second dose follow up and new cohort planned for June 2020. There is need to evaluate how HPV implementation and routinisation is being carried out, building up from the national introduction. This is particularly important given that three delivery platforms are being utilized nationally whereas only one was tested before and only in one district. Lessons from the HPV vaccine experience so far help the EPI to further improve on HPV vaccination as routinisation progresses. In addition, despite the demonstrable cost-effectiveness of the HPV vaccine, its routinisation and the ability to reach high coverage are dependent on the ability of the national immunisation programme to finance the costs associated with HPV vaccination into the routine immunisation system. There are concerns about the financial impact of HPV on the overall immunisation programme budget. The financial impact of HPV needs to be assessed carefully, and options for financing HPV explored.

This evaluation is being conducted to assess EPI learning from previous experiences in vaccine introduction and routinisation. It will seek to answer how HPV vaccine can be routinized for the best coverage whilst optimizing resource use as far as the different delivery platforms are concerned.

**Questions**

Introduction:

Please tell us briefly about your organisation and your role as far as HPV vaccination is concerned.

***Evaluation question 4:* To what extent is the routinisation of HPV being implemented as planned from a programmatic and financial perspective and what lessons can be drawn for effective integration of HPV into the national immunization programme?**

1. *Programmatically, what lessons can be drawn from HPV national introduction and implementation thus far to inform future programming for adequate routinisation of HPV?*
   1. What learning from other vaccine delivery or introductions, if any, have been applied in introducing HPV nationally? (Probe: leadership and governance, planning, coordination, social mobilisation, financing, technical support, HR, skills and knowledge, M&E, supervision, etc.)
   2. What learning from the HPV demo has been applied in introducing HPV nationally? (Probe: leadership and governance, planning, coordination, social mobilisation, financing, technical support, HR, skills and knowledge, M&E, supervision, etc.)
   3. What has been novel/innovative about the HPV national introduction compared to the HPV demo or other vaccine introductions? Probe: How well have these strategies worked?
   4. What have been the challenges and successes for HPV introduction so far across the three delivery platforms (School, outreach, health facility)?

- Which delivery platform(s) are working better, and which ones are not working well?
- How have the challenges been handled?
  1. What can be done differently in future based on current learning? Probe: have these been factored into the upcoming June 2020 immunisation and if so how?
  2. How well integrated is HPV into the immunisation programme in general? Probe: What can be done to further improve HPV integration into the EPI?

1. *To what extent are concerns about financial sustainability affecting the way the national introduction of HPV is being done?* Probe: differences across the three delivery platforms
   1. In view of perceived high costs of HPV vaccine and sustainability concerns, what measures were put in place to militate against this during national introduction?
   2. How well has this worked?
   3. Could anything be done differently for better results in future as far as financial sustainability is concerned?
   4. Are other, non-traditional modes of funding EPI being considered for HPV?
2. *What is the impact of HPV national introduction on the immunisation budget? (*for evaluation questions c and d)
   - How has HPV vaccine affected the immunisation budget?
   - What are the most costly components of HPV vaccination and what makes them so? Probe: What strategies can be put in place to cater for this cost in future or to reduce it?
   - In terms of the way the national introduction was carried out what worked well in terms of cost-saving measures and why?
   - Is the impact of HPV on the budget sustainable in the long run?
   - Have any other parts of the EPI suffered/received less funds or priority as a result of introduction of HPV vaccine?
3. How integrated is HPV vaccine delivery into adolescent health interventions and what are the implications of this financially and for sustainability? Would integration be beneficial or harmful for HPV vaccination? E.g. use of youth peer educators, integration into SRHR activities and/or HIV/AIDS activities
   1. What adolescent health services are offered in the health facilities?
   2. What has been done, if anything, to integrate HPV vaccination into adolescent health services?
   3. How well has this worked? What are the challenges and successes?
   4. What are the implications of such integration on the cost of delivering HPV vaccine?
   5. If nothing has been done to integrate HPV vaccine, what are the challenges in doing so?
